# Dengue in Timor-Leste during the COVID-19 phenomenon

**DOI:** 10.3389/fpubh.2023.1057951

**Published:** 2023-08-22

**Authors:** Zito Viegas da Cruz, Afonso Lima Araujo, Alexis Ribas, Choosak Nithikathkul

**Affiliations:** ^1^Master of Science Program in Tropical Health Innovation, Faculty of Medicine, Mahasarakham University, Muang, Mahasarakham, Thailand; ^2^Health Statistics Information Ministry of Health (MoH), Dili, Timor-Leste; ^3^Parasitology Section, Department of Biology, Healthcare and Environment, Faculty of Pharmacy and Food Science, Institut de Recerca de la Biodiversitat, University of Barcelona, Barcelona, Spain; ^4^Tropical Health Innovation Research Unit, Faculty of Medicine, Mahasarakham University, Muang, Mahasarakham, Thailand

**Keywords:** dengue, COVID-19, mapping, GIS, Timor-Leste

## Abstract

Dengue is a significant public health problem in mostly tropical countries, including Timor-Leste. Dengue continues to draw attention from the health sector during the COVID-19 phenomenon. Therefore, the goal of this study is to evaluate the dengue incidence rate in comparison with the COVID-19 cumulative number and associated dengue risk factors, including the fatality rate of dengue infection in each municipality during the COVID-19 phenomenon in Timor-Leste, by applying the data processing program in Geographic Information Systems (GIS). A descriptive study using GIS was performed to provide a spatial-temporal mapping of dengue cases. Secondary data, which were sourced from the Department of Health Statistics Information under the Ministry of Health Timor-Leste, were collected for the period during the COVID-19 outbreak in 2020–2021. These data were grounded at the municipal (province) level. Quantum GIS and Microsoft Excel were used to analyze the data. During the COVID-19 outbreak (2020–2021), dengue spread nationwide. It was found that there was an increase in municipalities with high dengue cases and cumulative COVID-19 numbers. The high number of dengue cases associated with the COVID-19 cumulative number found in municipalities with an urban characteristic and in terms of severity, dengue fever (DF) is most commonly reported with a total of 1,556 cases and is followed by dengue hemorrhagic fever (DHF) and dengue shock syndrome (DSS). Most cases were reported in the months of the monsoon season, such as December, January, and March. Dengue GIS mapping helps understand the disease's presence and dynamic nature over time.

## 1. Introduction

Dengue has become a serious public health problem, commonly in tropical countries, due to its compatibility with mosquito habitats such as those in Timor-Leste (1, 2). In addition to dengue as a disease of the tropical climate that is common, several other neglected tropical diseases are very common in Timor-Leste, such as scabies and impetigo (3), malaria, tuberculosis (4, 5), and rheumatic heart disease (6). In regards to all of this, much effort has been made in Timor-Leste to deal with it, but the situation still attracts the attention of the public health sector.

The novel Corona Virus 2019 (COVID-19), caused by the SARS-CoV-2 virus, has become a pandemic with a growing number of cases globally (7). With the rapid spread of COVID-19, global health systems are experiencing critical challenges in preventing infections, identifying and managing COVID-19 cases, and ensuring effective strategies to protect public health (8, 9). Afterward, in Timor-Leste, the first positive COVID-19 case was identified on March 21, 2020 (10). From that point on, the government monitored the situation, and to protect its health system, Timor-Leste established the “Centro Integrado de Gestão de Crise” (CIGC: Integrated Centre for Crisis Management) in March 2020 (11).

As Timor-Leste grapples with the threat of a resurgence of the coronavirus, an alarming spike in seasonal dengue fever has simultaneously presented a new challenge for health workers. Since a large proportion of health resources, such as human resources, laboratory testing, hospitals, and epidemiological monitoring, are dedicated to COVID-19, other diseases, such as dengue infection, are experiencing substantial delays in diagnosis and treatment. In addition, during the COVID-19 pandemic, health workers in dengue-endemic areas or those caring for patients who have recently visited the area must include dengue fever and COVID-19 in the differential diagnosis of acute febrile illness due to Clinically, the manifestations of dengue virus (DENV) infection can range from mild-acute undifferentiated febrile illness to classical dengue fever (DF), dengue hemorrhagic fever (DHF), and dengue shock syndrome (DSS) (12). Dengue fever (DF) is an acute febrile illness that causes symptoms such as bone or joint and muscular pains, headaches, leukopenia, and a rash. Dengue hemorrhagic fever (DHF) has four major clinical manifestations: severe fever, hemorrhage, often with hepatomegaly, and, in severe cases, circulatory failure. In dengue shock syndrome (DSS), some of the infected individuals may develop hypovolemic shock due to severe plasma leakage. When the temperature reaches 37.5–38°C, a decrease in platelet count leads to leakage of plasma, subsequent shock, fluid accumulation with respiratory distress, critical bleeding, organ impairment, cardiorespiratory failure, and cardiac arrest (13). Therefore, there is a tendency for misdiagnosis between SARS-CoV-2 virus and dengue virus (DENV) infections (14, 15). Further, if a dengue infection is co-infected with SARS-CoV-2, this co-occurrence will be highly concerning, s both conditions may potentially lead to fatal outcomes (16).

Dengue fever has become more widespread in the past few decades around the world. Based on one projection, more than 390 million people are infected with the dengue virus (DENV) annually, with 96 million people manifesting clinical symptoms (with any severity of disease) (17, 18). Dengue-endemic areas have faced the additional public health and socioeconomic impact of the ongoing COVID-19 pandemic (19). Timor-Leste's situation is no exception. Authorities in Timor-Leste have reported a surge of dengue cases since late 2021, at unusually high levels compared to previous years before the COVID-19 pandemic (20, 21). There were 1,451 reported cases and 10 deaths with a case fatality rate of 0.7% in 2020 and 901 cases and 11 deaths with a case fatality rate of 1.2% in 2021. In January 2022 alone, 1,286 cases were reported, with 20 fatalities, for a case fatality rate of 1.6% (22). This worrisome spike in case counts is partly explained by a shift in national policies for recording and reporting dengue fever to Ministries of Health (MoH) and the World Health Organization (WHO).

In this regard, several studies have found that dengue infection is associated with climate change, geographical, environmental, and sociodemographic conditions, including the expansion of the reach of its main vector (the *Aedes* mosquito), rapid and unplanned urbanization, the movement of people for trade, tourism, or because of natural disasters, and vulnerability in public health and vector control programs (23–28). Furthermore, an outbreak of dengue infection will be more severe if a climate disaster occurs, at the same time, such as a flood or hurricane, because it will increase the outbreak of other infectious diseases such as cholera, diarrhea, and so on during the COVID-19 phenomenon (29).

Thus, within the context of global environment changes, both local and global change scenarios exert significant impacts on environmental and physical phenomena, as well as health informatics issues. Some patients who reached the critical stage of these effects extended them from individual to global scales, encompassing a broad spectrum of influence. Thus, health information on neglected tropical and non-communicable outbreaks among all levels and international health organizations is still necessary for new trends and technological application approaches to develop prevention and control strategies, and integrating multidisciplinary networks is still considered essential (30–36). Therefore, in this study, we conducted a descriptive observational study to evaluate the dengue incidence rate in comparison with the COVID-19 cumulative number and associated dengue risk factors, including the fatality rate of dengue infection in each municipality during the COVID-19 phenomenon in Timor-Leste, by applying the data processing program in Geographic Information Systems (GIS) as a computer-based system, consisting of hardware and software that facilitate the capture, retrieval, management, manipulation, analysis, and display of the spatially geo-referenced data (37, 38), is to evaluate the present state of dengue in Timor-Leste to better understand the existing efforts and difficulties, as well as to find possibilities for development through innovative suggestions for all stakeholders.

Mapping disease is vital to supporting dengue surveillance because this tool allows the user to customize the disease picture across time in a specific space or population (39). This disease dynamics map also provides policymakers with information. Knowing the presence of disease and developing appropriate disease prevention strategies require spatial information.

## 2. Materials and methods

### 2.1. Study area

This study is defined as a descriptive and observational study. Timor-Leste occupies a land area of 15,007 km2 in the eastern part of the island of Timor and is located between 8.1 and 9.5°S and 125.0 and 127.3°E, including the small enclave of Oecussi between 9.2 and 9.5°S and 124.1 and 124.5°E located in the western half of the island within West Timor. The country is divided into 13 municipalities. The population of Timor-Leste was 1,340,434 (40). Dili, the capital city, is the only major population center with over 300,000 people, with no other towns with more than 200,000 people. The majority of the population lives in rural areas and practices subsistence farming (26).

### 2.2. Data source and data collection

We used secondary data sets that were derived from a related institution and aggregated by municipality (province). Dengue morbidity and mortality were collected from the dengue monthly report of Health Statistics Information Systems (HIS/EIS) under the Ministry of Health Timor-Leste (MoH.TL) office during 2020–2021. Dengue positives in this study were described as every patient who tested positive for dengue fever (DF), dengue hemorrhagic fever (DHF), and dengue shock syndrome (DSS) by laboratory testing in the health facilities in each of the 13 municipalities before it was reported to the HIS/EIS department, along with the data of COVID-19 cumulative incidence from the first positive case reported on March 21, 2020, to December 2021, including the data of population density for each year during the COVID-19 phenomenon. Further, we used data recorded entirely in Health Statistics Information (HIS/EIS) under the Ministry of Health Timor-Leste (MoH.TL). We described variables in this research to explore the distribution of dengue cases in Timor-Leste during the COVID-19 phenomenon. Dengue cases, which are served as the incidence rate per municipality by year, are compared with the COVID-19 cumulative number reported, the population density for each municipality from each year, the distribution of dengue cases according to severity, and the comparison among dengue cases reported monthly, including the dengue fatality rate in each municipality during the COVID-19 pandemic.

### 2.3. Analysis data

**Figure 1:**
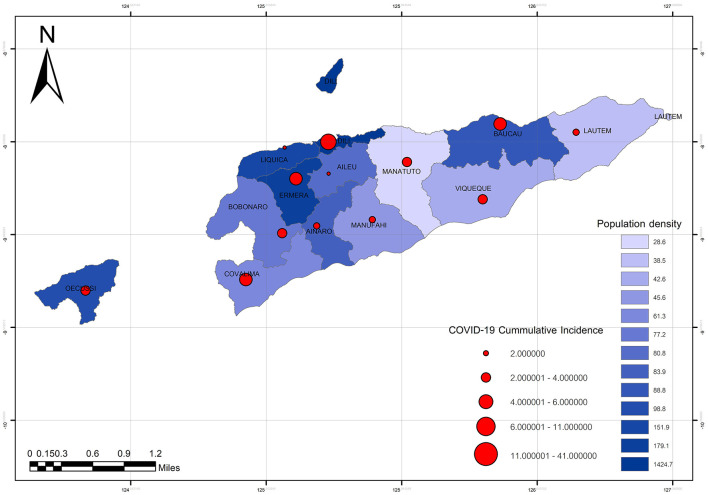
The COVID-19 cumulative incidence in comparison with the population density in each of the 13 municipalities of Timor-Leste.

An analysis was performed using Microsoft Excel for trend analysis. Furthermore, using secondary data, trend analysis was used to forecast future dengue incidence rates. Spatiotemporal mapping was generated using Quantum GIS (QGIS). It is used in data analysis when data are collected across both space and time. The dengue phenomenon at a certain location and time is described at a certain location and time, such as at the municipality level, and the time of incidence rate presented. Thus, the Geographic information on COVID-19 Cumulative incidence in Timor-Leste is based on population density in each of the 13 municipalities (Figure 1) and is necessary in evaluating dengue incidence during the COVID-19 phenomenon. Furthermore, the trend analysis result is shown on the map of the area.

## 3. Result

### 3.1. The trend in dengue incidence

**Figure 2:**
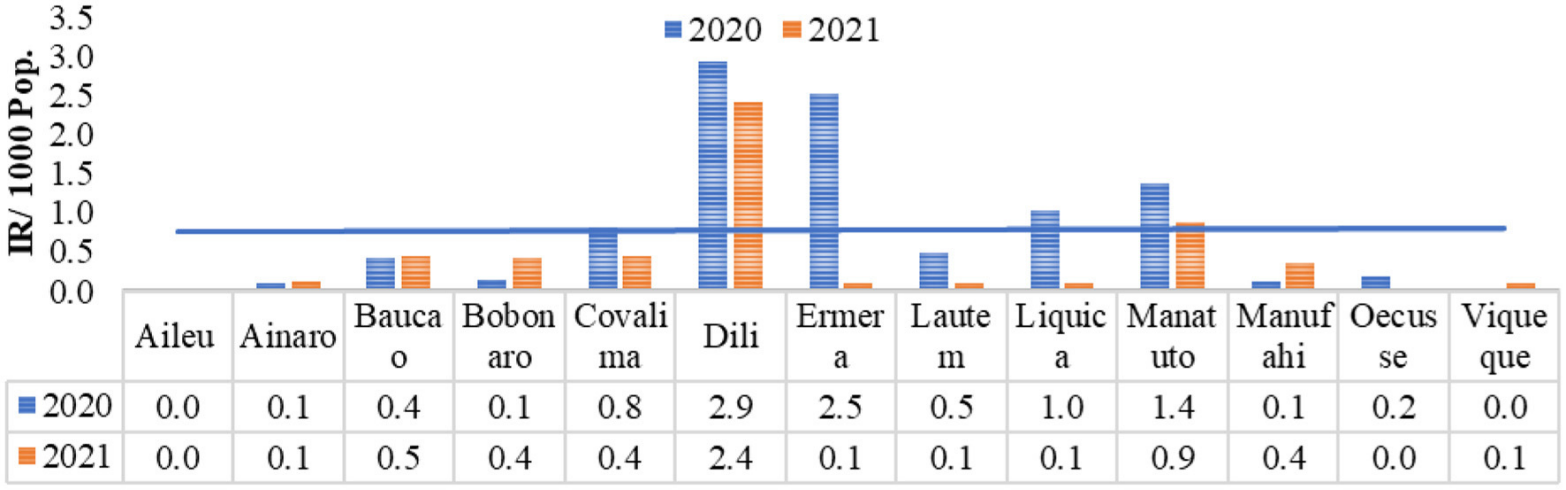
Dengue incidence rate per municipality in Timor-Leste during the COVID-19 phenomenon by 2020–2021.

Dengue case incidence in each municipality seems to be declining during the COVID-19 phenomenon from 2020–2021. This can be seen from the linear line in Figure 2.

### 3.2. Spatiotemporal and cumulative number of COVID-19 cases mapping

**Figure 3:**
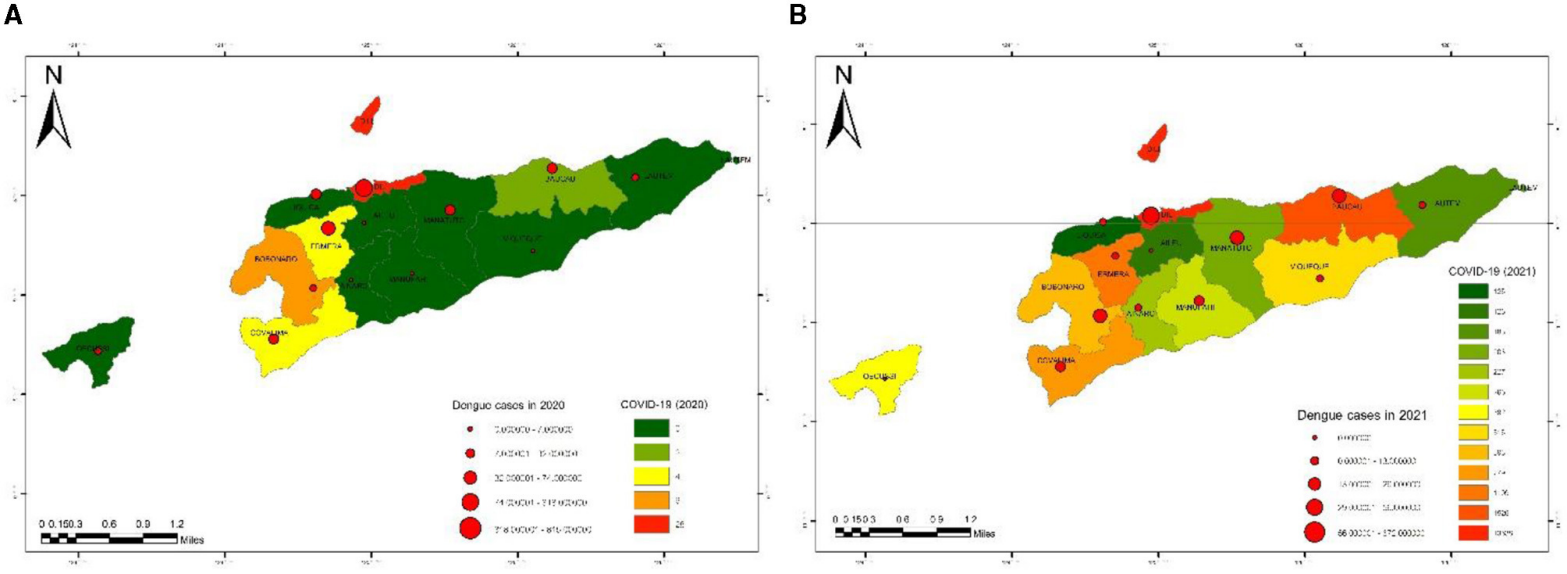
Spatiotemporal mapping of dengue incidence rates by municipalities in Timor-Leste in comparison with COVID-19 cases in 2020 and 2021 **(A, B)**.

During the COVID-19 pandemic, it was simultaneously unexpected that dengue infection spread across Timor-Leste's 13 municipalities. It is predominantly found in urban areas such as Dili municipality (the capital city), Ermera, and Baucau (41, 42). However, two other municipalities, such as Manatuto and Liquica, have a direct geographical border area with the capital city, Dili Municipality^1^, and the majority of them are classified as having a high and medium incidence rate (IR), and the other eight municipalities are not directly bordered with the capital city, which is classified as having a low incidence rate (IR) during the last two years of each 2020 and 2021. Furthermore, the result of this study suggests that in each of the municipalities with a high or medium dengue incidence rate in the last 2 years, 2020 and 2021, there was a correlation with the COVID-19 cumulative case number reported (Figure 3).

### 3.3. Spatiotemporal and population density mapping

**Figure 4:**
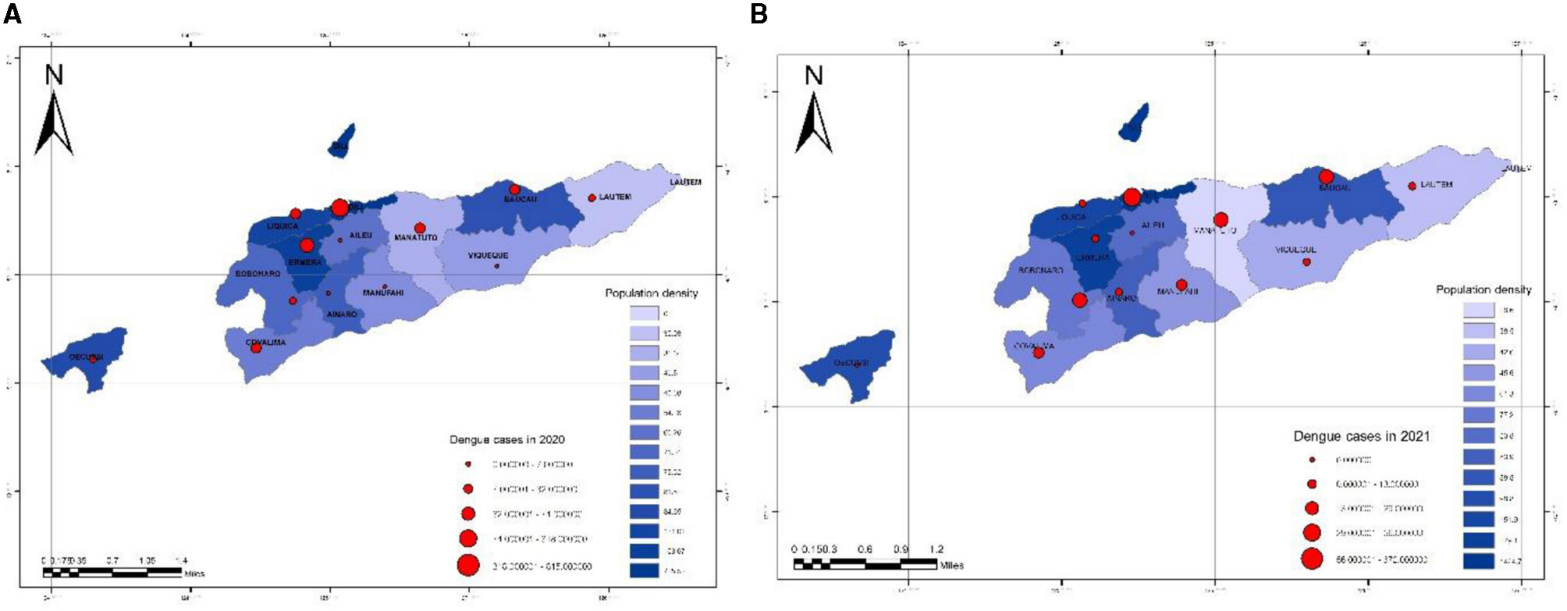
Spatiotemporal mapping of dengue incidence rate by municipalities in Timor-Leste in comparison with population density in 2020 and 2021 **(A, B)**.

During the COVID-19 period (2020–2021), there were dengue cases in Timor-Leste, which were associated with sociodemographic conditions, particularly with the population density in each of the 13 Municipalities placed between 2,589.940000 and 15,404.39000 population density per square kilometer. Dili municipality, the Capital city of Timor-Leste, has the highest population density, followed by Ermera and Baucau municipalities, and Aileu, Manatuto, and Manufahi municipalities, which have predicated the lowest population density (Figure 4).

### 3.4. Distribution of dengue cases according to severity

**Figure 5:**
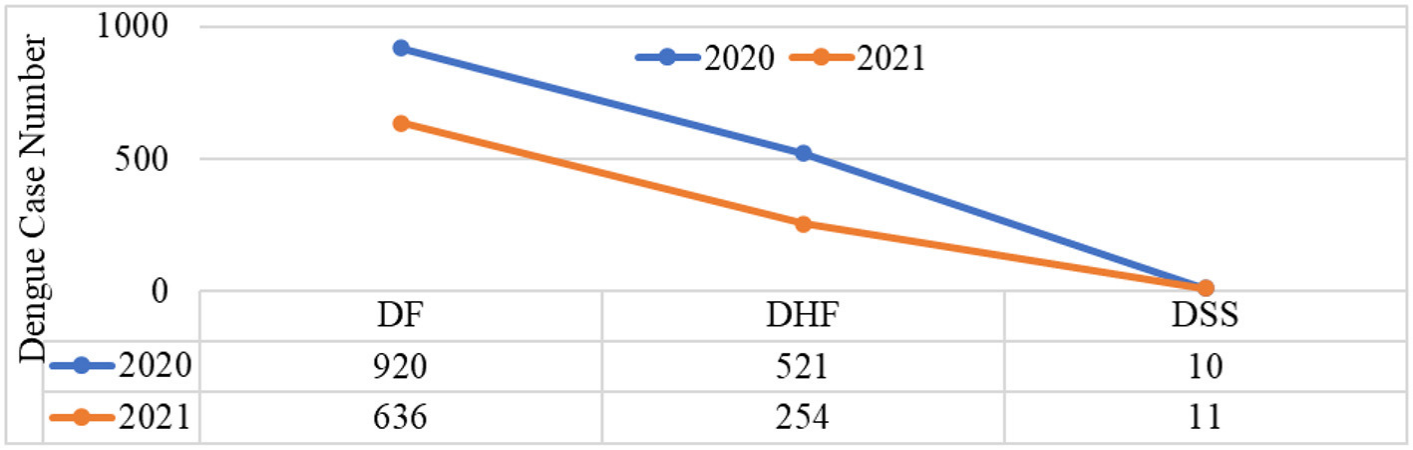
Classification of dengue cases according to severity.

According to the report from the Health Statistic Information Department under the Ministry of Health (MoH) of Timor-Leste, dengue cases were reported in order of severity, such as dengue fever (DF), dengue hemorrhagic fever (DHF), and dengue shock syndrome (DSS), based on symptoms and laboratory results (number of hemoglobin, leukocyte, thrombocyte, hematocrit, and rapid diagnostic tests (RDTs)]. The results show that dengue fever (DF) was most frequently reported in each year; in 2020, there were 920 cases reported, and in 2021, there were 636 cases reported out of all the total dengue cases reported during the COVID-19 phenomenon in the whole country. Dengue hemorrhagic fever (DHF) and dengue shock syndrome (DSS) were less than half of the total dengue cases reported each year during the period of the COVID-19 pandemic (Figure 5).

### 3.5. Monthly dengue cases report

**Figure 6:**
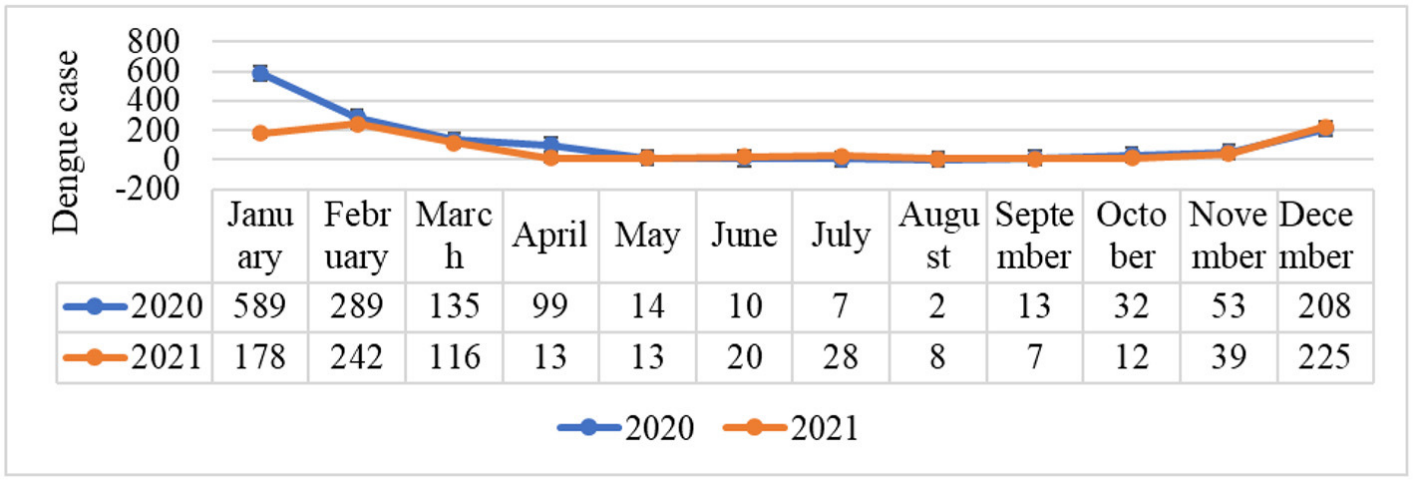
Dengue cases were compared every month in 2020 and 2021.

In order to reported cases monthly during the time of the COVID-19 outbreak in Timor-Leste in 2020–2021, there were the most reported dengue cases in December–March (Figure 6), which is the rainy season in Timor Leste (43).

### 3.6. The dengue fatality rate in each municipality

**Figure 7:**
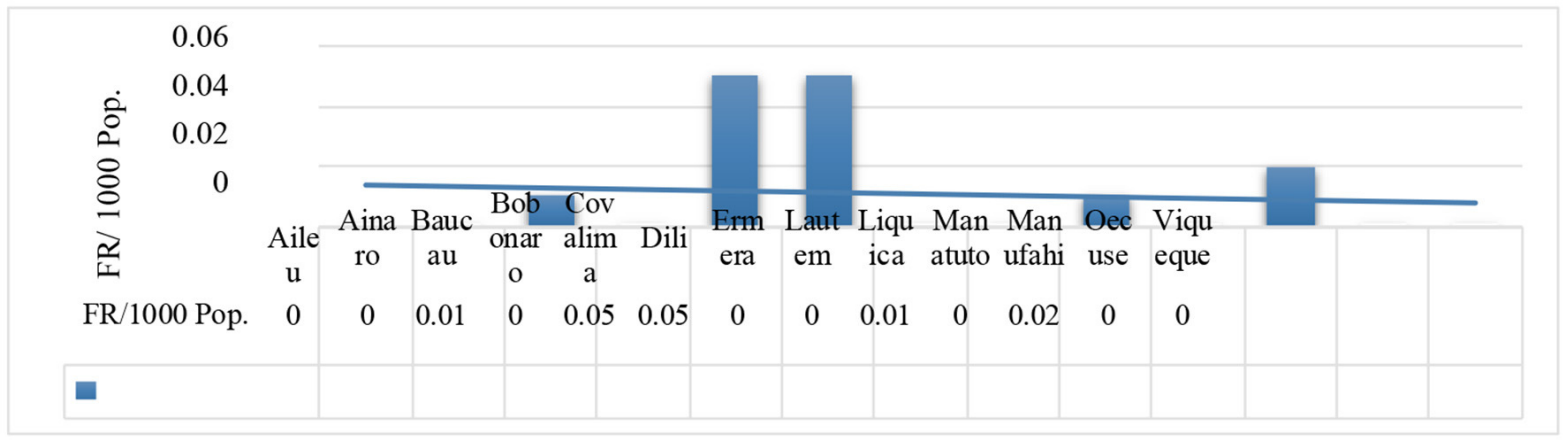
The fatality rate due to dengue cases in each municipality from 2020 to 2021.

During the period of the COVID-19 phenomenon in Timor-Leste, there were reported 21 mortality cases with a Fatality Rate (FR) of 0.02/1000 population, and of those, the Dili municipality, the capital city, had the most reported mortality cases with a total of 15, with a Fatality Rate (FR) of ~0.05/1,000 population, followed by the Covalima Municipality (Figure 7).


R/ Colima=Covalima Municipality

## 4. Discussion

This is the first study to explore dengue incidence in Timor-Leste during the COVID-19 phenomenon. There was no evidence of spatial clustering of dengue risk after accounting for the covariates, indicating that variability in COVID-19 cumulative cases, precipitation, and sociodemographic conditions such as population density in an area, explain much of the spatiotemporal dynamics of the disease, particularly during the COVID-19 pandemic. This is similar to findings from studies in other parts of the world (16, 29, 44, 45). Furthermore, during the COVID-19 pandemic worldwide, all countries felt the impact, including Timor-Leste. This is a new challenge for health workers.

The current policy of dengue eradication in Timor-Leste, under the Ministry of Health, is implemented using the guidelines outlined in the Bi-regional Dengue Strategy (2008–2015) (WHO Southeast Asia and Western Pacific regions) (46). This involves a multi-pronged approach based on case management through early detection and diagnosis; vector control via spraying; source reduction activities in the community, including distributing larvicides and fumigating malathion (a mosquito adulticide) in residential quarters; mobilizing communities and volunteers to clean up water containers; and environmental education on prevention and surveillance (22). Dengue is mostly diagnosed based on clinical findings. While current guidelines recommend the use of rapid diagnostic tests (RDTs), they are not widely used. Dengue cases are subject to obligatory notification, and cases reported are collated by the Department of Epidemiological Surveillance at the Ministry of Health (26). However, due to the COVID-19 pandemic, implementing all of these was a new challenge for program managers and the community in general.

During the COVID-19 period, dengue cases in Timor-Leste kept fluctuating, with the highest incidence rate (IR) per 1,000 population in 2020 at 2.94 and the lowest incidence rate (IR) per 1,000 population at 0.00 in 2021. Public health measures that restricted the movement of people and closed schools, universities, and offices to contain COVID-19 transmission unexpectedly led to a significant reduction in the reported numbers of dengue cases in Timor-Leste. This result is in accordance with a study by Surendran et al. (47). However, the number of reported dengue cases during the last 2 years of the COVID-19 pandemic is substantially higher compared to the same periods in previous years before the COVID-19 pandemic (20, 21). Furthermore, dengue in Timor-Leste is not unexpected, but the significant daily increase in the number of dengue cases and the rate of hospitalization in the current outbreak is unusual (22).

Further, this study shows that the dengue cases reported by each municipality in Timor-Leste had a significant association with the presented COVID-19 cumulative case number. However, in consideration of this result, we assumed the existence of an uncertain diagnosis among these two disorders due to Timor-Leste's fragile healthcare system (48), particularly a lack of clinical laboratory systems. Furthermore, the coronavirus disease 2019 (COVID-19) has non-specific clinical and laboratory characteristics (49), which might be similar to other viral infections, including dengue. Therefore, it is a challenging context for certain diagnostics in Timor-Leste. This is consistent with preview studies by Ratnarathon et al. and Yan et al., which reported that dengue and coronavirus disease 2019 are difficult to distinguish because they have shared clinical and laboratory features (45, 50). Furthermore, to avoid misdiagnosis among these two disorders, we have to make an accurate differential diagnosis.

Population density is well-known to have contributed to dengue transmission in this study. This result, in accordance with previous research in Indonesia's Sleman district, found that dengue incidence is associated with population density, and high dengue incidence has occurred in areas of high population density (51). Further, in accordance with preview studies by Arifin et al. (39), Romeo-Aznar et al. (52), and Nagarani et al. (53), they report that unplanned population growth, mainly in the urban area, is responsible for the emergence of slum areas with insufficient sanitation that serve as mosquito breeding sites.

According to the severity of the dengue cases, the most reported is dengue fever (DF), followed by dengue hemorrhagic fever (DHF) and dengue shock syndrome (DSS). It was noted that, due to the limited resources and the COVID-19 outbreak, almost all the dengue fever (DF) reported was clinically suspected, which is not diagnostic in any way (22). Due to the similarity of the clinical manifestations shared by COVID-19 and dengue fever (DF), this has led to diagnostic challenges in Timor-Leste as a dengue-endemic area and raised concerns. This finding is consistent with the preview study by Harapan et al., in which it was reported that the similar clinical manifestations shared by COVID-19 and dengue fever (DF) had raised concerns, especially in dengue-endemic countries with limited resources, leading to diagnostic challenges (14). Further, a study by Pastor Bandeira et al. reported that due to the similarity of dermatological signs and symptoms presented in both disorders, a previously healthy patient with COVID-19 was mistakenly diagnosed with dengue fever (DF) due to a skin rash (54). Therefore, in accordance with this uncertain diagnosis, this is a highly concerning public health problem in Timor-Leste.

The monthly reported dengue cases during the COVID-19 phenomenon were the dengue cases most reported in the wettest and hottest months of the year (December to April) in Timor-Leste (43) and are likely to be associated with vector dynamics. This condition is because heavy rains can cause flooding and water stagnation in several risk places, which potentially facilitates vector population growth by providing water for mosquito breeding sites, mainly the *Aedes* sp. mosquitoes, and this phenomenon can contribute to other infection diseases being more complicated. This finding was in accordance with preview studies by Wangdi et al. and Hu et al., who reported that dengue cases were associated with precipitation in a dengue-endemic area (26, 55). Furthermore, in accordance with a study in Bangladesh by Rahman et al., the COVID-19 pandemic, dengue epidemic, and climate change revealed the high possibility of exacerbating the impact of the COVID-19 pandemic if there is a climatic hazard such as floods or cyclones (29).

In accordance with the case Fatality Rate (FR), Dili (the capital city) and Covalima municipalities were higher than eleven other municipalities, and the preliminary discussion with the program manager indicated that because of the COVID-19 impact, such as poor access to health facilities, the mortality probably happened due to the prolonged duration of admission to the hospital where people become ill. Some patients who reached the critical stage of the disease quickly experienced deterioration. Consequently, in the context of Dili municipality, there was an increase in reported cases of dengue-related mortality. Dili has the highest reported population density among the other 12 municipalities and is the center of all of the sectors (56). This finding is consistent with preview studies by Díaz-Quijano and Waldman, who discovered that, in addition to the age of endemicity, it is associated with several other risk factors such as environment (rainfall), demographics (population density), socioeconomics, and biology (circulating serotypes) that affect and contribute to the alarming increase in dengue mortality (57). Meanwhile, a study on dengue and COVID-19, overlapping epidemics, found that both conditions may potentially lead to fatal outcomes, especially in patients with chronic comorbidities, and overlapping infections and co-occurrence may increase the number of patients requiring intensive care and mechanical ventilation (16).

### 4.1. Limitation

There are a number of limitations to this study. The dengue data were collected through passive surveillance of people with fever cases attending a health unit and, for the most part, being clinically diagnosed, which overlooks the contribution of asymptomatic dengue fever cases, which can be confused with the similarity of COVID-19 patients' symptoms and signs. In this study, we did not find any data on dengue patients co-infected with COVID-19.

### 4.2. Strengths

Furthermore, the strengths of this study are useful for policymakers to prioritize areas of action, mainly the dengue incidence during the COVID-19 period in Timor-Leste compared with the previous years before the COVID-19 pandemic. The quality of the dengue surveillance system may vary over time and between locations, as the awareness of dengue among medical professionals and public health workers may have increased as a result of the WHO's initiatives to train and sensitize the aforementioned health workers. It is possible that the national surveillance system operates more effectively in and around the capital than in remote areas. This might have contributed to the low estimated disease burden outside of Dili and Ermera municipalities. In addition, considering this study's result, the next study proposes to assess the association between dengue and climatic variation as part of climate change. It seeks analytical evidence of a relationship between population knowledge, attitude, and practice (KAP) in dengue prevention and control; human population growth (rapid and unplanned urbanization); movement of people for trade, tourism, or forced by natural disasters; and vulnerability in public health and vector control programs and dengue cases. Dengue prevention efforts must be sustained by health authorities through integrated surveillance.

## 5. Conclusion

Dengue incidence and fatality rates in Timor-Leste during the COVID-19 period are prevalent in urban areas and associated with COVID-19 cumulative case numbers, population density, and precipitations. Because of the lack of access to health care during the COVID-19 pandemic and the fragility of health care systems, unplanned urbanization, including environmental conditions (rainy season) that can cause flooding and water stagnation in several risk places and potentially facilitate mosquito breeding sites, were significant predictors of dengue incidence in Timor-Leste. In addition, according to severity, dengue fever (DF) is higher.

Nowadays, the Geographic Information System (GIS) is applied to discovering risk areas for epidemic diseases, including dengue infection globally (58, 59), and is one of the most effective tools for dengue surveillance in an area. This calls for public health actions and other sectors to mitigate future dengue risk factors from environmental and geographical conditions. In addition, the Geographic Information System (GIS) is a highly useful tool for Timor-Leste as a young country and as an innovation tool to contribute to dengue surveillance, among other infectious diseases.

Further studies are needed to define if these risk areas are maintained over time, while similar studies can be applied to other neglected tropical diseases such as malaria, tuberculosis, and filariasis, which affect both municipalities in Timor-Leste and other Southeast Asian countries.

## Author contributions

CN contributed to the conception and design, had full access to all of the data in the study and took responsibility for the integrity of the data, accuracy of the data analysis, supervision, and obtaining partial funding. ZC wrote the manuscript and acquisition of partial funding. AA provided secondary data on dengue cases and COVID-19 cumulative case numbers. AR revised English grammar and writing format. All authors contributed to the acquisition or interpretation of the data, critical revision of the manuscript for important intellectual content, and the final approval of the manuscript for publication.

## Footnotes

^1^gov. 10 Timor-Leste 2016 demographic qnd health survey (2016).

## References

[1] Rahman MS Pientong C Zafar S Ekalaksananan T Paul RE Haque U. Mapping the spatial distribution of the dengue vector Aedes aegypti and predicting its abundance in northeastern Thailand using machine-learning approach. One Heal. (2021) 13:100358. 10.1016/j.onehlt.2021.100358 34934797 PMC8661047

[2] Molyneux N da Cruz GR Williams RL Andersen R Turner NC. Climate change and population growth in Timor Leste: Implications for food security. Ambio. (2012) 41:823–40. 10.1007/s13280-012-0287-0 22569843 PMC3492559

[3] Korte LM Bowen AC Draper ADK Davis K Steel A Teodora I. Scabies and impetigo in Timor-Leste: a school screening study in two districts. PLoS Negl Trop Dis. (2018) 12:9–15. 10.1371/journal.pntd.0006400 29852002 PMC5997349

[4] Manel Yapabandara AMG Do Rosario M De Fatima Mota M Sarmento R Bosco JD Wickremasinghe R. From malaria control to elimination within a decade: lessons learned from Timor Leste, a newly independent country. Malar J. (2020) 19:1–12. 10.1186/s12936-020-03162-3 32127001 PMC7055025

[5] Pengpid S Peltzer K. Knowledge, attitudes, and practices regarding tuberculosis in Timor-Leste: results from the demographic and health Survey 2016. J Prev Med Public Heal. (2019) 52:115–22. 10.3961/jpmph.18.170 30971078 PMC6459764

[6] Davis K Remenyi B Draper AD Dos Santos J Bayley N Paratz E. Rheumatic heart disease in Timor-Leste school students: an echocardiography-based prevalence study. Med J Aust. (2018) 208:303–7. 10.5694/mja17.00666 29642817

[7] Rehman MFU Fariha C Anwar A Shahzad N Ahmad M Mukhtar S. Novel coronavirus disease (COVID-19) pandemic: a recent mini review. Comput Struct Biotechnol J. (2021) 19:612–23. 10.1016/j.csbj.2020.12.033 33398233 PMC7773542

[8] Magson NR Freeman JYA Rapee RM Richardson CE Oar EL Fardouly J. Risk and protective factors for prospective changes in adolescent mental health during the COVID-19 pandemic. J Youth Adolesc. (2021) 50:44–57. 10.1007/s10964-020-01332-9 33108542 PMC7590912

[9] Atchison CJ Bowman L Vrinten C Redd R Pristerà P Eaton JW. Perceptions and behavioural responses of the general public during the COVID-19 pandemic: A cross-sectional survey of UK adults. medRxiv. (2020) 1–21. 10.1101/2020.04.01.20050039 PMC7783373 33397669

[10] Niha MA Draper AD Viegas Od de Araujo RM Joao JC da Silva E. The epidemiology of the COVID-19 pandemic in the small, low-resource country of Timor-Leste, January 2020 - June 2022. Commun. Dis. Intell. (2023) 47. 10.33321/cdi.2023.47.1 36654501

[11] Governo O. Número Extraordinário. (2020). p. 1–6.

[12] Habib G Hoen B Tornos P Thuny F Prendergast B Vilacosta I., Guidelines on the prevention, diagnosis, and treatment of infective endocarditis (new version 2009). Eur. Heart J. (2009) 30:2369–413. 10.1093/eurheartj/ehp285 19713420

[13] Wang W-H Urbina AN Chang MR Assavalapsakul W Lu P-L Chen Y-H. Dengue hemorrhagic fever – A systemic literature review of current perspectives on pathogenesis, prevention and control. J Microbiol Immunol Infect. (2020) 53:963–78. 10.1016/j.jmii.2020.03.007 32265181

[14] Harapan H Ryan M Yohan B Abidin RS Nainu F Rakib A. Covid-19 and dengue: Double punches for dengue-endemic countries in Asia. Rev Med Virol. (2021) 31:1–9. 10.1002/rmv.2161 32946149 PMC7536968

[15] Nacher M Douine M Gaillet M Flamand C Rousset D Rousseau C. Simultaneous dengue and COVID-19 epidemics: difficult days ahead? PLoS Negl Trop Dis. (2020) 14:1–8. 10.1371/journal.pntd.0008426 32797035 PMC7428060

[16] Cardona-Ospina JA Arteaga-Livias K Villamil-Gómez WE Pérez-Díaz CE Bonilla-Aldana DK Mondragon-Cardona Á. Dengue and COVID-19, overlapping epidemics? An analysis from Colombia. J Med Virol. (2021) 93:522–7. 10.1002/jmv.26194 32558962 PMC7323437

[17] Bhatt S Gething PW Brady OJ Messina JP Farlow AW Moyes CL. The global distribution and burden of dengue. Nature. (2013) 496:504–7. 10.1038/nature12060 23563266 PMC3651993

[18] Guzman MG Gubler DJ Izquierdo A Martinez E Halstead SB. Dengue infection. Nat Rev Dis Prim. (2016) 2:1–26. 10.1038/nrdp.2016.55 27534439

[19] Wilder-Smith A. Dengue during the COVID-19 pandemic. J Travel Med. (2021) 28:1–2. 10.1093/jtm/taab183 34850050 PMC8690170

[20] Sánchez-González L Venuto M Poe S Major CG Baskara L Abdiyeva S. Dengue virus infections among peace corps volunteers in Timor-Leste, 2018–2019. Am J Trop Med Hyg. (2021) 104:2202–9. 10.4269/ajtmh.21-0020 33901000 PMC8176509

[21] T. D. Outbreak. Information Bulletin the Situation (2019). p. 2–5. Available online at: https://www.ifrc.org/docs/Appeals/IBTLPD~29042019.pdf

[22] World Health Organization. Dengue - Timor-Leste. (2022). Available online at: https://www.who.int/emergencies/disease-outbreak-news/item/dengue—timor-leste

[23] Rahman M Shammi M. Since January 2020 Elsevier has Created a COVID-19 Resource Centre With Free Information in English and Mandarin on the Novel Coronavirus COVID- 19. The COVID-19 Resource Centre is Hosted on Elsevier Connect, the Company' s Public News and Information. (2020).

[24] Sulistyawati S Fatmawati F. GIS For Dengue Surveillance: A Systematic Review (2020).

[25] Erandi K Perera S Mahasinghe AC. Analysis and forecast of dengue incidence in urban Colombo, Sri Lanka. Theor Biol Med Model. (2021) 7:1–19. 10.1186/s12976-020-00134-7 33413478 PMC7791698

[26] Wangdi K Clements ACA Du T Nery SV. Spatial and temporal patterns of dengue infections in Timor-Leste, 2005–2013. Parasit Vectors. (2018) 11:1–9. 10.1186/s13071-017-2588-4 29301546 PMC5755460

[27] Gananalatha E. An investigation into spatial vulnerable factors for dengue epidemics using GIS in the Matara district in Sri Lanka. Int. Res. J. Med. Sci. (2017) 5:1–8.

[28] Jat MK Mala S. Application of GIS and Statistical Modelling for Dengue Fever Surveillance in Delhi, India (2016). 10.15224/978-1-63248-114-6-17

[29] Rahman MM Bodrud-Doza M Shammi M Islam AR Khan AS. COVID-19 pandemic, dengue epidemic, and climate change vulnerability in Bangladesh: scenario assessment for strategic management and policy implications. Environ Res. (2021) 192:110303. 10.1016/j.envres.2020.110303 33069704 PMC7561529

[30] Toemjai T Thongkrajai P Nithikathkul C. Factors affecting preventive behavior against leptospirosis among the population at risk in Si Sa Ket, Thailand. One Heal. (2022) 14:100399. 10.1016/j.onehlt.2022.100399 35686145 PMC9171525

[31] Nakbun S Thongkrajai P Nithikathkul C. Risk factors for *Opisthorchis viverrini* infection in Nakhon Phanom, Thailand, where the infection is highly endemic. Asian Biomed. (2018) 12:45–51. 10.1515/abm-2018-0030

[32] Soncharoen P Jongthawin J Nithikathkul C. Influent factor toward based helminth infections among of Thai-Cambodian Border in Phusing District, Sisaket Province, Thailand. Int Geoinformatics J. (2022) 18:71–86. 10.52939/ijg.v18i5.2375

[33] Kanjaras P Bumrerraj S Seng R Noradee S Nithikathkul C. Geospatial analysis and modeling of melioidosis prevention and control in Si Sa Ket Province, Thailand. Int Geoinformat J. (2023) 19:57–65. 10.52939/ijg.v19i1.2501

[34] Nithikathkul C Trevanich A Wongsaroj T Wongsawad C Reungsang P. Health informatics model for helminthiasis in Thailand. J Helminthol. (2017) 91:528–33. 10.1017/S0022149X16000614 27666946

[35] Nithikathkul C Wongsaroj T Buntilov V Limsomboon J. Geographic information system of fish-borne parasitic Zoonoses metacercaria from water reservoirs under his Majesty's recommended project, Phitsanulok, Thailand. Int J Geoinformat. (2012) 8:53–7.

[36] Wongpituk K Kalayanarooj S Nithikathkul C. Geospatial analysis of DHF surveillance model in Si Sa Ket Province, Thailand using geographic information system. Int Geoinformatics J. (2020) 16:97–104.

[37] Definitions O. Lecture 3. What is GIS? Geographic information systems. Inf Syst. (1997).

[38] Maguire DJ. An overview and definition of GIS. In:Maguire DJ Goodchild MF Rhind DW, editors. Geographical Information Systems: Principles and Applications, Vol. 1. Hoboken: Wiley (1991). p. 9–20.

[39] Arifin NF Adi MS Suwondo A. Spatial and Temporal Determinantsfor Dengue Haemorrhagic Fever: A Descriptive Study in Tanjungpinang City, Indonesia Spatial and Temporal Determinantsfor Dengue Haemorrhagic Fever: A Descriptive Study in Tanjungpinang City, Indonesia. (2018). 10.9790/0853-1610133438

[40] URT. Preliminary Results of Population and Housing Census (2022).

[41] dos Ferreira ES. Launch of the Main Results of the 2015 Census of Population and Housing (2016).

[42] Ito M Takasaki T Kotaki A Tajima S Yuwono D Rimal HS. Molecular and virological analyses of dengue virus responsible for dengue outbreak in East Timor in 2005. Jpn J Infect Dis. (2010) 63:181–4. 10.7883/yoken.63.181 20495269

[43] CSIRO and SPREP. ‘NextGen' Projections for the Western Tropical Pacific: Current and Future Climate for Kiribati (2021).

[44] Huang L Xiao G Chen H Niu X Fu X Xu H. Geographical clusters of dengue outbreak in Singapore during the Covid-19 nationwide lockdown of 2020. Sci Data. (2022) 9:1–8. 10.1038/s41597-022-01666-y 36071062 PMC9451123

[45] Ratnarathon AC Pongpirul K Pongpirul WA Charoenpong L Prasithsirikul W. Potential dual dengue and SARS-CoV-2 infection in Thailand: a case study. Heliyon. (2020) 6:e04175. 10.1016/j.heliyon.2020.e04175 32542206 PMC7280131

[46] World Health Organization. 2020 Dengue. Dengue Southeast Asia. (2020). 41 p. Available online at: https://www.who.int/publications/i/item/dengue-bulletin-vol-41?sequence=1&isAllowed=y

[47] Surendran SN Nagulan R Sivabalakrishnan K Arthiyan S Tharsan A Jayadas TTP. Reduced dengue incidence during the COVID-19 movement restrictions in Sri Lanka from March 2020 to April 2021. BMC Public Health. (2022) 22:1–10. 10.1186/s12889-022-12726-8 35209890 PMC8866919

[48] Howitt R de Jesus GA Araujo F Francis J Marr I McVean M. Screening and triage at healthcare facilities in Timor-Leste during the COVID-19 pandemic. Lancet Respir Med. (2020) 8:e43. 10.1016/S2213-2600(20)30183-1 32333858 PMC7176383

[49] Guan W-J Ni Z-Y Hu Y Liang W-H Ou C-Q He J-X. Clinical characteristics of coronavirus disease 2019 in China. N Engl J Med. (2020) 382:1708–20. 10.1056/nejmoa2002032 32109013 PMC7092819

[50] Yan G Lee CK Lam LTM Yan B Chua YX Lim AYN. Covert COVID-19 and false-positive dengue serology in Singapore. Lancet Infect Dis. (2020) 20:536. 10.1016/S1473-3099(20)30158-4 32145189 PMC7128937

[51] Sulistyawati S Sukesi TW Mulasari SA. Spatiotemporal mapping of dengue cases in Sleman district, Indonesia year 2014-2017. Int J Community Med Public Health. (2019) 6:971–5. 10.18203/2394-6040.ijcmph20190579

[52] Romeo-Aznar V Picinini Freitas L Gonçalves Cruz O King AA Pascual M. Fine-scale heterogeneity in population density predicts wave dynamics in dengue epidemics. Nat Commun. (2022) 13:1–9. 10.1038/s41467-022-28231-w 35194017 PMC8864019

[53] Dom NC Ahmad AH Latif ZA Ismail R. Integration of GIS-based model with epidemiological data as a tool for dengue surveillance. EnvironmentAsia. (2017) 10:135–46. Available online at: www.tshe.org/ea/index.html

[54] Pastor Bandeira I Sordi Chara B Meneguzzi de Carvalho G Magno Gonçalves MV. Diffuse skin rash in tropical areas: Dengue fever or COVID-19? An Bras Dermatol. (2021) 96:85–7. 10.1016/j.abd.2020.10.001 33281008 PMC7670906

[55] Hu W Clements A Tong S Williams G Mengersen K. Spatial patterns and socioecological drivers of dengue fever transmission in queensland, Australia. Environ Health Perspect. (2012) 120:260–6. 10.1289/ehp.1003270 22015625 PMC3279430

[56] Ministry of Planning and Strategic. The Project for Study on Dili Urban Master Plan in the Democratic Republic of Timor-Leste (2016).

[57] Díaz-Quijano FA Waldman EA. Factors associated with dengue mortality in Latin America and the Caribbean, 1995-2009: an ecological study. Am J Trop Med Hyg. (2012) 86:328–34. 10.4269/ajtmh.2012.11-0074 22302870 PMC3269288

[58] Rogers DJ Randolph SE. Studying the global distribution of infectious diseases using GIS and RS. Nat Rev Microbiol. (2003) 1:231–7. 10.1038/nrmicro776 15035027 PMC7096846

[59] Hay SI Omumbo JA Craig MH Snow RW. Earth observation, geographic information systems and *Plasmodium falciparum* malaria in sub-Saharan Africa. Adv Parasitol. (2000) 47:173–4. 10.1016/S0065-308X(00)47009-0 10997207 PMC3164801

